# Prognostic nomogram in patients with right-sided colon cancer after colectomy: a surveillance, epidemiology, and end results–based study

**DOI:** 10.3389/fonc.2024.1330344

**Published:** 2024-03-14

**Authors:** Tiantian Qin, Chenyue Yu, Yuying Dong, Mingming Zheng, Xiaoxuan Wang, Xuning Shen

**Affiliations:** ^1^ Department of Cardio-Thoracic Surgery, Jiaxing First Hospital, Affiliated Hospital of Jiaxing University, Zhejiang, China; ^2^ Department of Gastroenterological Surgery, Jiaxing First Hospital, Affiliated Hospital of Jiaxing University, Zhejiang, China; ^3^ Department of Emergency Surgery, Jiaxing First Hospital, Affiliated Hospital of Jiaxing University, Jiaxing, Zhejiang, China

**Keywords:** nomogram, overall survival, prognosis, right-sided colon cancer, SEER

## Abstract

**Objective:**

This study aimed to develop and validate a nomogram for predicting overall survival (OS) in patients undergoing surgery for right-sided colon cancer (RCC).

**Methods:**

We collected 25,203 patients with RCC from the Surveillance, Epidemiology, and End Results (SEER) database and randomly divided them into 7:3 training and internal validation set. Utilizing the Cox proportional hazards regression model, we constructed a nomogram based on prognostic risk factors. Furthermore, for external validation, we retrospectively followed up with 228 patients from Jiaxing First Hospital and assessed and calibrated the nomogram using the C-index and calibration curves.

**Results:**

After identifying independent prognostic factors through univariate and multivariate analyses, a nomogram was developed. The c-index values of this nomogram differed as follows: 0.851 (95% CI: 0.845-0.857) in the training set, 0.860 (95% CI: 0.850-0.870) in the internal validation set, and 0.834 (95% CI: 0.780-0.888) in the external validation set, indicating the model’s strong discriminative ability. Calibration curves for 1-year, 3-year, and 5-year overall survival (OS) probabilities exhibited a high level of consistency between predicted and actual survival rates. Furthermore, Decision Curve Analysis (DCA) demonstrated that the new model consistently outperformed the TNM staging system in terms of net benefit.

**Conclusion:**

We developed and validated a survival prediction model for patients with RCC. This novel nomogram outperforms the traditional TNM staging system and can guide clinical practitioners in making optimal clinical decisions.

## Introduction

By 2020, an estimated 19,292,789 new cases of cancer were reported globally. Among these cases, colorectal cancer (CRC) ranked as the third most common cancer, accounting for approximately 10.0% of the total (1, 2). According to the sources, colorectal cancer led to 935,173 deaths, representing 9.4% of the total cancer-related mortality. This makes colorectal cancer the second leading cause of cancer-related deaths, following only lung cancer. Predictions suggest that by 2030, the incidence of colorectal cancer is expected to significantly increase, with an estimated 2.2 million new cases and approximately 1.1 million related death (3).

Colorectal cancer stands apart from other malignant tumor sites due to its distinct anatomical distribution. The colon and rectum can be anatomically categorized into three main segments: the right colon (including the cecum, ascending colon, hepatic flexure of the colon, and transverse colon), the left colon (encompassing the descending colon, sigmoid colon, and splenic flexure of the colon), and the rectum (encompassing the junction of the rectum and sigmoid colon). These distinct anatomical regions exhibit differential sensitivity to carcinogens due to variations in embryology and physiology. Consequently, tumors arising in these segments may demonstrate disparate pathogenic mechanisms, varying diagnostic sensitivity, distinct clinicopathological characteristics, and differing prognostic outcomes (4). As a result, some researchers advocate for the consideration of colon cancer as comprising two or more distinct disease types (5). Recent investigations have revealed a shifting incidence trend in colorectal cancer towards the right colon (6). Notably, in China, the incidence rate of right colon cancer surpasses that of rectal cancer. Data analysis spanning from 1980 to 1990 demonstrates an increase in the incidence of right colon cancer from 10.9% to 15.2% in China. Furthermore, relative to left colon cancer, right colon cancer is associated with a less favorable prognosis (7).

Presently, the preeminent framework utilized for forecasting cancer survival and guiding clinical decisions is the American Joint Committee on Cancer (AJCC) staging guidelines (8). However, it’s noteworthy that the prognostic guidelines established by AJCC solely incorporate parameters such as tumor size, lymph node involvement, and metastasis status, inadvertently overlooking additional variables that possess the potential to significantly influence a patient’s postoperative prognosis. It is imperative to acknowledge that these guidelines primarily extrapolate outcomes for population rather than tailoring predictions for individual patient.in previous studies on CRC, several predictive models have been established (9, 10), but models specific to RCC are scarce. In many cancers, nomograms have demonstrated superiority over the traditional TNM staging system (11, 12). Clinicians can estimate the cumulative effects of all prognostic factors for a given patient and predict the probabilities of 1-year, 3-year, and 5-year survival rates from the nomogram (13). The primary objective of this study is to develop and validate a nomogram tailored for RCC, combining multiple indicators to predict postoperative survival outcomes for RCC patients.

## Methods

### Patients and selection criteria

In this study, we extracted data from Surveillance, Epidemiology, and End Results (SEER) Program (www.seer.cancer.gov), SEER*Stat Database: Incidence - SEER Research Plus Data, 18 Registries, Nov 2020 Sub (2000 - 2018). Between 2010 and 2015, we diagnosed 451,241 patients with RCC. Based on the inclusion and exclusion criteria, we ultimately selected 25,203 eligible patients. Patients were randomly assigned to an internal validation set (n = 7,561) or a training set (n = 17,642). The inclusion and exclusion criteria for the external validation set were the same as those used for the training set.

The inclusion and exclusion criteria were the same in the training and validation set. The inclusion criteria were (1) year of diagnosis between 2010 and 2015; (2) primary site code C18.0, C18.2, C18.3, or C18.4; (3) histologically confirmed diagnosis; (4) adenocarcinoma (histology codes 8140–8147, 8210, 8211, 8220, 8221, and 8260 - 8263), mucinous adenocarcinoma (histology codes 8480, 8481, and 8490); and (5) no history of another malignant tumor (sequence number: 1 primary only; first malignant primary indicator: yes). The exclusion criteria were (1) age < 18 years, (2) death or no follow-up within 30 days, and (3) other variables were unknown or missing from the database.

Ethical approval is not required for this article, as all data from the SEER database are obtained using publicly available methods. Participants involved in external validation have already received ethical approval from our institution (Ethics No. LS2021-KY-367).

### Include variables and processing

This study included a total of 17 variables, encompassing demographic information, tumor characteristic details, and treatment information.

Demographic information comprised age at diagnosis, gender, and race. Tumor-specific details consisted of primary site, histologic type, grade, derived AJCC T stage (7th ed), derived AJCC N stage (7th ed), derived AJCC M stage (7th ed), summary stage, preoperative carcinoembryonic antigen level (CEA), regional lymph nodes removed (LN), liver metastasis, lung metastasis, brain metastasis, and bone metastasis. Treatment details included postoperative chemotherapy status. Additionally, the patients’ vital status and survival time in months were incorporated.

Given that age is a continuous variable in the SEER database, this study classified ages using 10-year intervals: <31, 31-40, 41-50, 51-60, 61-70, 71-80, 81-90, and >90 years. LN were categorized as 1-3 and ≥4. Race were categorized as White, Black, Asian/Pacific Islander, and other races. Patients’ tumor primary sites were categorized as cecum, ascending colon, hepatic flexure, and transverse colon. Tumor grade was categorized as stages I (well-differentiated), II (moderately differentiated), III (poorly differentiated), and IV (undifferentiated), and tumor histologic types included adenocarcinoma and mucinous adenocarcinoma. TNM staging was based on the 7th edition of the American Joint Committee on Cancer guidelines, classifying primary tumor extent (T1, T2, T3, T4a, and T4b), lymph node involvement (N0, N1a, N1b, N1c, N2a, and N2b), and distant metastasis (M0, M1a, and M1b), while summary stage classified tumor spread as local, regional, or distant. This study’s follow-up initiation point was the diagnosis date of RCC, with overall survival (OS) as the endpoint, representing the time interval from diagnosis to patient death.

### Construction of the nomogram

The patients from the SEER database were randomly divided into training and validation dataset in a 7:3 ratio. A univariate Cox proportional hazards regression analysis was conducted, and factors with statistical significance (*P* < 0.05) were included in the multivariate Cox regression analysis to determine independent prognostic impact factors. For each variable, the corresponding 95% Confidence Interval (CI) and Hazard Ratio (HR) were calculated (14). All independent prognostic factors (*P* < 0.05) from the multivariate Cox regression analysis were integrated. Utilizing LASSO regression analysis and optimal subset regression analysis, factors selected were combined with the results from the multivariate Cox proportional hazards analysis to identify the prognostic factors to be included in the nomogram.Based on these independent prognostic factors, we employed statistical software (R 4.1.1, http://www.rproject.org/) to establish a nomogram for predicting the probabilities of 1-year, 3-year, and 5-year postoperative overall survival (OS) for RCC patients.

### Calibration and validation of the nomogram

Concordance index (C-index) and calibration curves are commonly used to evaluate the performance and accuracy of the nomogram. The C-index values range between 0.5 and 1, positively correlating with the predictive capability of the model. When this value surpasses 0.7, it indicates a reliable discriminative ability of the model (15). For model validation, internal validation was performed using the validation set, external validation was conducted using cases collected at our institution, and calibration curves were generated using bootstrapping resampling.

The calibration curve is a line passing through the origin with a slope of 1. The higher the predictive calibration curve approaches the standard curve, the greater the predictive capacity of the nomogram. Decision curve analysis (DCA), a novel analytical technique, integrates all clinical consequences of a decision and quantifies the clinical utility of a predictive model (16).

Furthermore, DCA was employed to ascertain whether the nomogram is more accurate than the AJCC TNM staging system, aiming to further assess the benefits and advantages of the nomogram.

## Results

### Patient clinicopathologic characteristics

According to the inclusion and exclusion criteria, a total of 25,203 patients diagnosed with RCC were included from the SEER database. These patients were randomly divided in a 7:3 ratio, resulting in a training set (n = 17,642) and a validation set (n = 7,561). The training set was utilized for determining independent prognostic factors and constructing the nomogram, while the validation set was used for internal validation of the nomogram. The results indicated no significant differences between various indicators in the training and validation set (*P* > 0.05, as shown in **Table 1**), suggesting comparability between the two patient groups. The validation set’s patients could be utilized to verify the performance of the nomogram model. The follow-up period for all patients ranged from 1 to 107 months, with 7,864 patients having died during the follow-up period, resulting in a mortality rate of 31.2%.

**Table 1:** Clinicopathological characteristics of patients with right-sided colon cancer.

Variable	Training set (*n* = 17,642)	Validation set (n = 7,561)	*p*
Gender			0.779
Female	8,274 (46.8)	3,448 (45.6)	
Male	9,368 (53.2)	4,113 (54.3)	
Age (years)			< 0.001
<31	97 (0.5)	48 (0.6)	
31-40	414 (2.3)	183 (2.4)	
41-50	1,497 (8.4)	632 (8.3)	
51-60	3,296 (18.6)	1,367 (18.0)	
61-70	4,902 (27.7)	2,170 (28.6)	
71-80	4,429 (25.1)	1,913 (25.3)	
81-90	2,722 (15.4)	1,143 (15.1)	
>90	285 (1.6)	105 (1.3)	
Race			0.121
White	13,723 (77.7)	5,883 (77.8)	
Black	2,466 (13.9)	1,031 (13.6)	
Asian or Pacific Islander	1,313 (7.4)	592 (7.8)	
American Indian	140 (0.7)	55 (0.7)	
Site			< 0.001
Cecum	7,115 (40.3)	3,026 (40.2)	
Ascending colon	6,117 (35.1)	2,597 (34.3	
Hepatic flexure	1,398 (7.9)	672 (8.8)	
Transverse colon	2,952(16.7)	1,266 (16.7)	
Histologic type			< 0.001
COAD	15,449 (87.5)	6,640(87.8)	
MC	2,193 (12.5)	921 (12.2)	
Grade			< 0.001
Grade I	1,168 (6.6)	527 (6.9)	
Grade II	12,140 (68.8)	5,260 (69.5)	
Grade III	3,573 (20.2)	1,464 (19.3)	
Grade IV	761 (4.3)	310 (4.0)	
T.stage			< 0.001
T1	1,540 (8.7)	818 (7.2)	
T2	2,495 (14.1)	1856 (16.4)	
T3	10,126 (57.3)	6527 (57.8)	
T4a	2,127 (12.0)	2091 (18.5)	
T4b	1,354 (7.6)		
N.stage			< 0.001
N0	9,446 (53.5)	4,098 (54.1)	
N1a	2,065 (11.7)	861 (11.3)	
N1b	2,337 (13.2)	955 (12.6)	
N1c	252 (1.4)	126 (1.6)	
N2a	1,640 (9.2)	733(9.6)	
N2b	1,902 (10.7)	788 (10.4)	
M.stage			< 0.001
M0	14,934 (84.6)	6,429 (85.0)	
M1a	1,537 (8.7)	669 (8.8)	
M1b	1,171 (6.6)	463 (6.1)	
Summary.stage			< 0.001
Localized	6,429 (36.4)	2,850 (37.6)	
Regional	8,403 (47.6)	3,522 (46.5)	
Distant	2,810 (15.9)	1,189 (15.7)	
LN			< 0.001
<4	184 (1.0)	70 (1.0)	
≥4	17,458 (99.0)	7491 (99.0)	
Chemotherapy			< 0.001
Yes	7,105 (40.2)	3,015 (39.0)	
No	10,537 (59.8)	4,546 (61.0)	
Bone metastasis			< 0.001
Yes	68 (0.3)	19 (0.2)	
No	17,574 (99.7)	7,542 (99.8)	
Brain metastasis			< 0.001
Yes	22 (0.2)	7 (0.1)	
No	17,620 (99.8)	7,554 (99.9)	
Lung metastasis			< 0.001
Yes	378 (2.2)	474 (2.3)	
No	17,264 (97.8)	7,387 (97.7)	
Liver metastasis			< 0.001
Yes	1,866 (10.5)	803 (10.6)	
No	15,776 (89.5)	6,758 (89.4)	
CEA			< 0.001
Normal	10,443 (59.2)	4,524 (59.8)	
Positive	7,199 (40.8)	3,037 (40.2)	
Survival status			
Alive	12,140 (68.8)	5,199 (68.8)	
Dead	5,502(31.2)	2,362 (31.2)	

COAD, colon adenocarcinoma; MC, mucinous adenocarcinoma; RNE, regional nodes examined; RNP, regional nodes positive.

### Independent risk factors in the training set

After conducting univariate analysis using the COX proportional hazards regression model, the results indicated that the following factors significantly influenced postoperative overall survival (OS) with a significance level of *P* < 0.05: age, tumor differentiation grade, histologic type, T stage, N stage, M stage, summary stage, liver metastasis, brain metastasis, lung metastasis, bone metastasis, CEA level, and chemotherapy. On the other hand, gender and race showed no significant influence on postoperative OS (*P* > 0.05). The significant variables identified from the univariate analysis were included in the multivariate COX regression analysis, with a significance level of *P* < 0.05 defining them as independent prognostic factors. Through the multivariate COX analysis, it was found that gender, race, tumor site, and histologic type were not significantly correlated with postoperative overall survival (OS) (*P* > 0.05). The results of univariate and multivariate analyses are presented in **Table 2**.

**Table 2:** Univariate and Multivariate COX Regression Analysis.

Variable	Univariate Analysis		Multivariate Analysis	
	HR (95%CI)	*P*	HR (95%CI)	*P*
Gender				
Male	Reference			
Female	0.99 (0.94 - 1.05)	0.779		
Age (years)				
< 31	Reference		Reference	
31-40	0.65 (0.45-0.95)	0.026	0.71 (0.49-1.03)	0.075
41-50	0.72 (0.51-1.00)	0.053	0.84 (0.60-1.18)	0.322
51-60	0.72 (0.52-1.00)	0.049	0.92 (0.66-1.28)	0.632
61-70	0.69 (0.50-0.95)	0.025	0.99 (0.72-1.38)	0.969
71-80	0.79 (0.57-1.10)	0.160	1.30 (0.94-1.81)	0.116
81-90	1.23 (0.89-1.70)	0.217	2.02 (1.45-2.81)	< 0.001
>90	2.13 (1.49-3.05)	< 0.001	3.03 (2.11-4.35)	< 0.001
Race				
White	Reference			
Black	1.26 (1.18-1.36)	< 0.001		
Asian or Pacific Islander	0.93 (0.84-1.03)	0.185		
American Indian	0.90 (0.66-1.24)	0.532		
Site				
Cecum	Reference		Reference	
Ascending colon	0.77 (0.72-0.82)	< 0.001	0.96 (0.90-1.02)	0.169
Hepatic flexure	0.83 (0.75-0.92)	< 0.001	1.05 (0.94-1.16)	0.389
Transverse colon	0.82 (0.76-0.88)	< 0.001	1.01 (0.94-1.10)	0.731
Histologic type				
COAD	Reference		Reference	
MC	1.39 (1.29-1.49)	< 0.001	1.03 (0.98-1.09)	0.194
Histological grade				
Grade I	Reference		Reference	
Grade II	1.58 (1.38-1.82)	< 0.001	1.04 (0.96-1.13)	0.319
Grade III	2.93 (2.55-3.38)	< 0.001	1.12 (1.03-1.28)	0.008
Grade IV	3.39 (2.87-4.01)	< 0.001	1.14 (1.12-1.32)	0.016
T.stage				
T1	Reference		Reference	
T2	1.89 (1.50-2.37)	< 0.001	1.46 (1.16-1.83)	< 0.001
T3	5.25 (4.30-6.41)	< 0.001	1.98 (1.60-2.44)	0.001
T4a	13.88 (11.31-17.03)	< 0.001	3.01 (2.42-3.74)	< 0.001
T4b	16.10 (13.08-19.83)	< 0.001	3.38 (2.70-4.22)	< 0.001
N.stage				
N0	Reference		Reference	
N1a	2.28 (2.07-2.51)	< 0.001	1.51 (1.35-1.68)	< 0.001
N1b	3.66 (3.38-3.97)	< 0.001	2.19 (1.98-2.41)	< 0.001
N1c	3.36 (2.75-4.12)	< 0.001	1.82 (1.48-2.24)	< 0.001
N2a	5.54 (5.10-6.02)	< 0.001	2.70 (2.44-2.99)	< 0.001
N2b	9.31 (8.64-10.03)	< 0.001	3.64 (3.30-4.01)	< 0.001
M.stage				
M0	Reference		Reference	
M1a	6.43 (6.02-6.88)	< 0.001	1.75 (1.30-2.34)	< 0.001
M1b	9.94 (9.26-10.68)	< 0.001	2.26 (1.69-3.02)	< 0.001
Summary.stage				
Localized	Reference		Reference	
Regional	3.84 (3.51-4.20)	< 0.001	1.90 (1.69-3.02)	< 0.001
Distant	19.00 (17.34-20.82)	< 0.001	2.88 (2.13-3.90)	< 0.001
LN				
1-3	Reference		Reference	
≥4	0.64 (0.52-0.80)	< 0.001	0.67 (0.54-0.84)	< 0.001
Chemotherapy				
Yes	Reference		Reference	
No	1.91 (1.81-2.01)	< 0.001	1.62 (1.51-1.73)	< 0.001
Bone metastasis				
No	Reference		Reference	
Yes	8.71 (6.78-11.2)	< 0.001	1.53 (1.18-1.99)	0.012
Brain metastasis				
No	Reference		Reference	
Yes	7.00 (4.46-10.99)	< 0.001	1.38 (1.17-1.59)	0.016
Lung metastasis				
No	Reference		Reference	
Yes	5.86 (5.24-6.55)	< 0.001	1.13 (1.00-1.29)	0.046
Liver metastasis				
No	Reference		Reference	
Yes	6.69 (6.30 - 7.10)	< 0.001	1.52 (1.38-1.67)	< 0.001
CEA				
Normal	Reference		Reference	
Positive	2.91 (2.76 - 3.08)	< 0.001	1.44 (1.36-1.53)	< 0.001

HR, hazard ratio; CI, confidence interval; COAD, colon adenocarcinoma; MC, mucinous adenocarcinoma; RNE, regional nodes examined.

**Figure 1:**
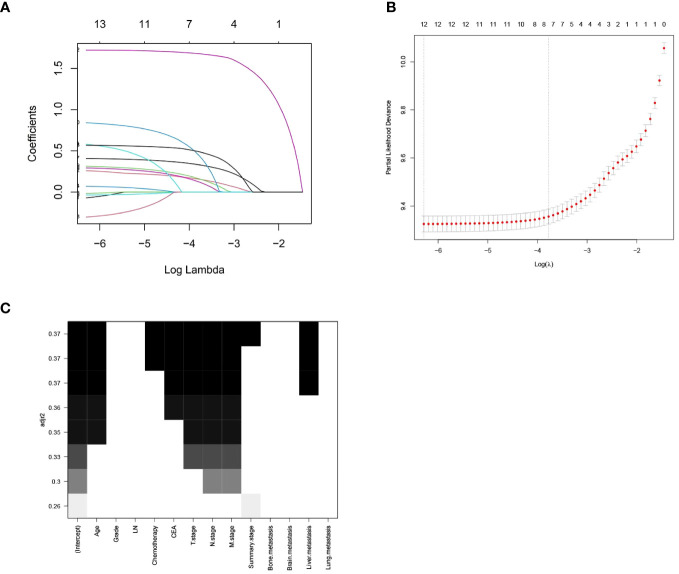
LASSO Regression Analysis and Optimal Subset Regression Analysis. **(A)** Distribution of LASSO coefficients for all variables of RCC. **(B)** 8 variables identified by LASSO analysis. **(C)** Optimal subset regression model selecting 8 variables.

After performing LASSO regression and best subset regression analyses (**Figure 1**), the variables tumor differentiation, number of regional lymph nodes removed, lung metastasis, brain metastasis, and bone metastasis were eliminated. Instead, the variables age, chemotherapy, CEA, T stage, N stage, M stage, summary stage, and liver metastasis were retained.

### Prognostic nomogram for OS

**Figure 2:**
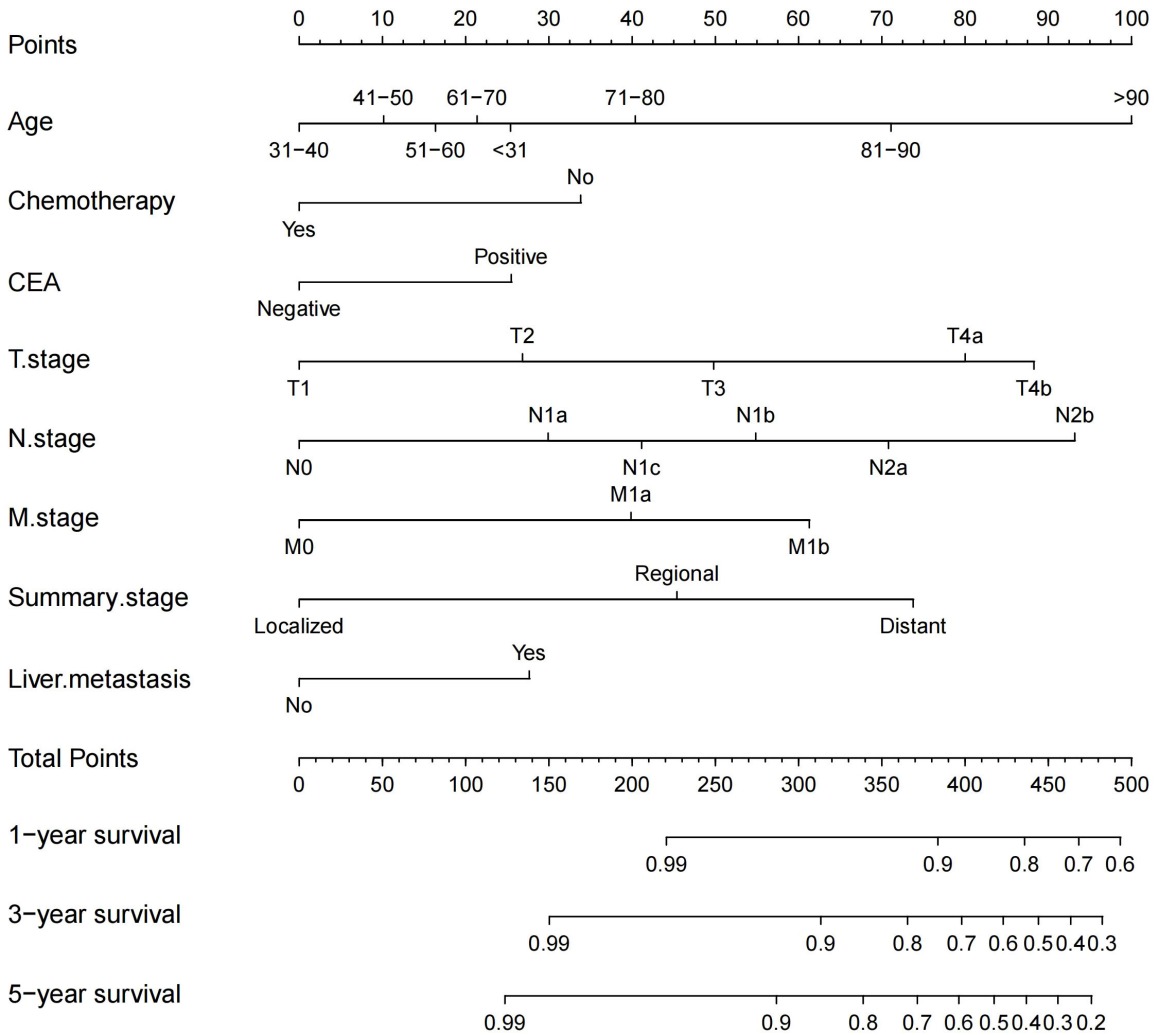
Nomogram for predicting 1-, 3-, and 5-year OS probabilities in patients with RCC after colectomy.

We constructed a traditional nomogram based on the results of the multiple regression and LASSO regression analyses mentioned earlier (**Figure 2**). The model incorporated age, chemotherapy, CEA, T stage, N stage, M stage, summary stage, and liver metastasis. The scores for each variable are shown in **Table 3**. The variables yielded total scores predicting 1-, 3-, and 5-year OS probabilities. By summing up the scores for each factor, a total score is obtained. This total score can be matched with the corresponding 1-year, 3-year, and 5-year OS coordinates at the bottom of the nomogram, providing the probability values for survival at these time points for RCC patients. Higher total scores indicate a worse prognosis.

**Table 3:** Scores of the variables.

Variable	Score	Variable	Score	Variable	Score
Age (years)		M.stage		N.stage	
<31	25	M0	0	N0	0
31-40	0	M1a	40	N1a	30
41-50	10	M1b	61	N1b	55
51-60	16	**T.stage**		N1c	41
61-70	21	T1	0	N2a	71
71-80	40	T2	27	N2b	93
81-90	71	T3	50	**CEA**	
>90	100	T4a	80	Normal	0
**Summary.stage**		T4b	88	Positive	25
Localized	0	**Chemotherapy**		**Liver metastasis**	
Regional	45	Yes	0	No	0
Distant	74	No	34	Yes	28

Specifically, age, N stage, and T stage are considered key factors influencing the scoring system. It is noteworthy that for individuals aged over 90 years, with N2b and T4b stages, their corresponding scores are 100, 93, and 88, respectively. Conversely, scores associated with CEA positivity, liver metastasis, and chemotherapy tend to be relatively lower, at 25, 28, and 34, respectively. For instance, a 73-year-old patient, undergoing chemotherapy, without liver metastasis but with CEA positive, and with a T4a, N1c, M0, and regional summary stage, accrues a total score of 206 according to the nomogram. This places the patient within the intermediate-risk category, with an estimated 5-year survival rate of approximately 56.75%.

**Figure 3:**
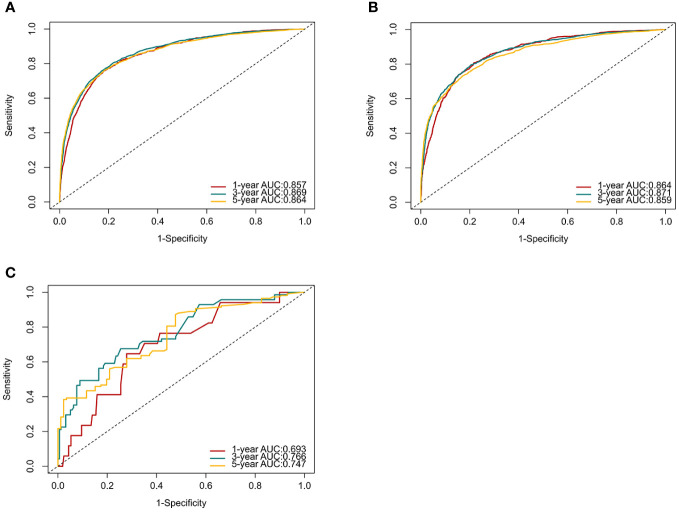
ROC curves and AUCs at 1, 3, and 5 years in the training set **(A)**, internal validation set **(B)** and the external validation set **(C)** were used to estimate the prognostic accuracy of the nomogram.

**Figure 4:**
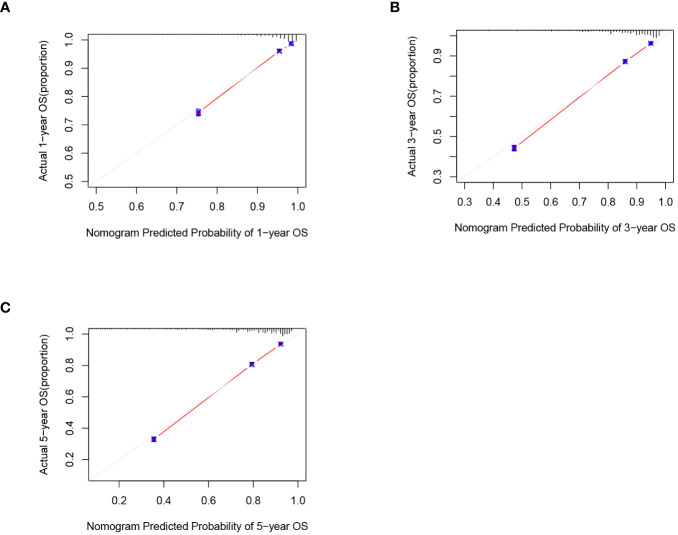
Calibration graphs forecasting the 1-, 3-, and 5-year overall survival (OS) of patients within the training set **(A)**, 1-year overall survival **(B)**, 3-year overall survival**(C)**. 5-year overall survival.

**Figure 5:**
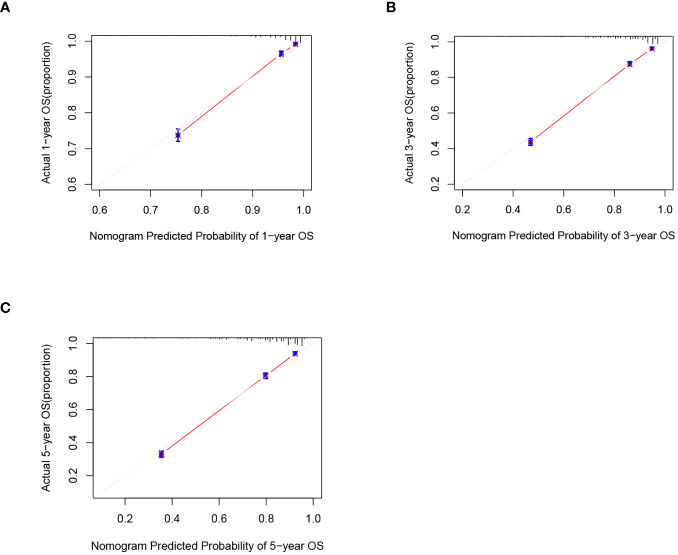
Calibration graphs forecasting the 1-, 3-, and 5-year overall survival (OS) of patients within the internal validation set **(A)**, 1-year overall survival **(B)**, 3-year overall survival **(C)**. 5-year overall survival.

**Figure 6:**
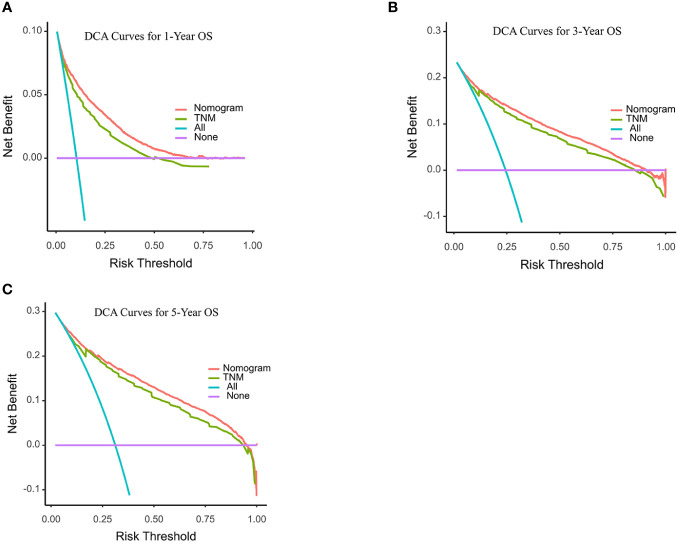
Decision curve analyses (DCA) of the nomogram and AJCC TNM staging system for 1-year **(A)**, 3-year **(B)**, and 5-year **(C)** overall survival. The x-axis represents the threshold probabilities, and the y-axis measures the net benefit. The horizontal line along the x-axis assumes that overall death occurred in no patients, whereas the solid purple line assumes that all patients will have overall death at a specific threshold probability. The Orange dashed line represents the nomogram. The green dashed line represents AJCC TNM staging system.

C-index and AUC values were used to evaluate the accuracy and discrimination of the nomogram. In the training set, the C-index of the nomogram for OS was 0.851 (95% CI: 0.845-0.857), and the 1-, 3-, and 5-year AUCs were 0857、0.869、0.724, respectively (**Figure 3A**). The C-index in the internal validation set was 0.860 (95% CI: 0.850-0.870), and the 1-, 3-, and 5-year AUCs were 0.864, 0.871, and 0.859, respectively (**Figure 3B**). To assess model performance internally, the time-dependent area under the receiver operating characteristic curve was calculated at different time-points. Calibration curves for the probability of postoperative OS at 1-year, 3-year, and 5-year (**Figures 4**, **5**) indicated that there was good consistency between the actual observation and the prediction. In contrast to the AJCC TNM staging approach, the decision curve analysis (DCA) exhibited a substantial rise in the net advantage for the novel nomogram graph, spanning a broad and feasible spectrum of threshold probabilities (**Figure 6**).

### External validation of the predictive accuracy of the nomogram for OS

**Figure 7:**
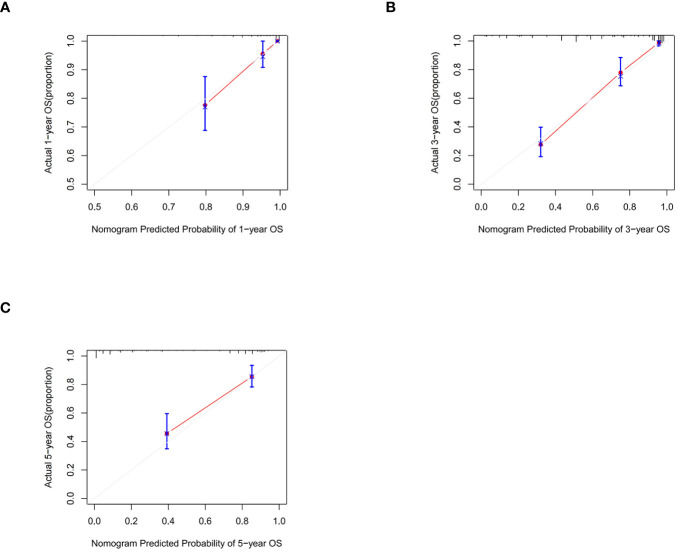
Calibration graphs forecasting the 1-, 3-, and 5-year overall survival (OS) of patients within the external validation set **(A)**, 1-year overall survival **(B)**, 3-year overall survival **(C)**. 5-year overall survival.

Following the same inclusion and exclusion criteria as the SEER database, a total of 228 cases of primary RCC patients who underwent surgery in the Department of Gastrointestinal Surgery at the First Hospital of Jiaxing from January 2014 to December 2017 were ultimately collected for external validation to further assess the predictive capability of the nomogram (**Table 4**). In the external Verification set, the C-index was 0.834(95%CI:0.780 - 0.888), and the 1-, 3- and 5-year AUCs were 0.693, 0.766, and 0.747 respectively (**Figure 3C**). The calibration curves for 1-year, 3-year, and 5-year survival (**Figure 7**) demonstrated a high level of agreement between predicted values and actual survival probabilities. These validation results indicate that the nomogram developed in this study exhibits a high level of accuracy and precision, making it suitable for predicting 1-year, 3-year, and 5-year overall survival in patients with right-sided colon cancer after surgery.

**Table 4:** External validation patient clinical characteristics information.

Variable	External validation (*n* = 228)	*p*
Age (years)		< 0.001
<31	3 (1.3)	
31-40	5 (2.1)	
41-50	20 (8.6)	
51-60	41 (17.8)	
61-70	72 (31.3)	
71-80	63 (27.3)	
81-90	24 (10.4)	
>90	0 (0.0)	
T.stage		< 0.001
T1	13(5.6)	
T2	12 (5.2)	
T3	173 (75.2)	
T4a	23 (10.0)	
T4b	7 (3.0)	
N.stage		< 0.001
N0	127 (55.2)	
N1a	22 (9.5)	
N1b	21 (9.1)	
N1c	34 (14.7)	
N2a	13 (5.6)	
N2b	11 (4.7)	
M.stage		< 0.001
M0	198(86.0)	
M1a	14 (6.0)	
M1b	16 (6.9)	
Summary.stage		< 0.001
Localized	22 (9.5)	
Regional	176 (76.5)	
Distant	30 (13.0)	
Chemotherapy		< 0.001
Yes	97 (42.1)	
No	131 (57.9)	
Liver metastasis		< 0.001
Yes	3 (1.3)	
No	225 (98.7)	
CEA		< 0.001
Normal	10,443(59.2)	
Positive	7,199(40.8)	
Survival status		
Alive	12,140(68.8)	
Dead	5,502(31.2)	

### Development and production of a web-based nomogram

To facilitate clinicians’ use of our Nomograms, we’ve created dynamic line graphs utilizing the “DynNom” package from R software. You can directly access it via the following https://tian1234.shinyapps.io/DynNomapp/. Once you input the predictor variables, the calculated survival probabilities can be easily displayed. It’s user-friendly and doesn’t require any permission or login credentials from clinicians.

### Risk stratification of the nomogram

**Figure 8:**
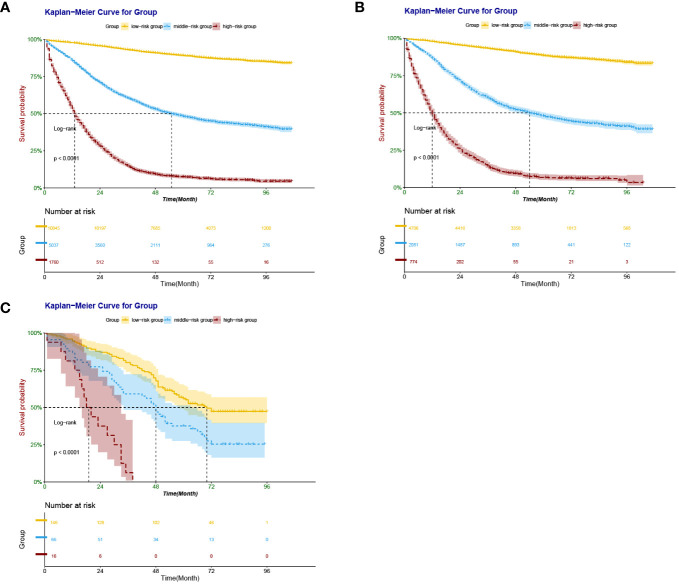
Kaplan–Meier survival curves derived from nomogram-based groups of patients with RCC after colectomy. The *p* value (<0.0001) was determined by the log-rank test. **(A)** Kaplan–Meier survival curves derived from nomogram-based groups of patients with RCC after colectomy in the training set. **(B)** Kaplan–Meier survival curves derived from nomogram-based groups of patients with RCC after colectomy in the internal validation set. **(C)** Kaplan–Meier survival curves derived from nomogram-based groups of patients with RCC after colectomy in the external validation set.

According to the X-Tile software, patients with scores <197, 198 - 313, and > 313 points were divided into low-risk, intermediate-risk, and high-risk groups, respectively. Training set: 10,838 low-risk cases (61.43%), 5,021 medium-risk cases (28.46%), and high-risk 1,783 cases (10.11%). Internal validation set: 4,713 low-risk cases (62.33%), 2,097 medium-risk cases (27.73%), 751 cases (9.94%) were at high risk. External validation set: 146 cases of low risk (64.03%), 66 cases of medium risk (28.95%), and 16 cases of high risk (7.02%). Kaplan-Meier survival analysis for each risk group showed that the OS of the low-risk group was significantly better than that of the intermediate-risk group and high-risk group (*P <*0.001) (**Figure 8**), further validating the nomogram-based model to predict risk scores for patients with right-sided colon cancer has important clinical implications.

## Discussion

The diagnosis and prognosis of colorectal cancer remain central and intricate topics in the medical field. Given the high incidence and mortality rate of colorectal cancer, numerous clinical research centers have pivoted towards harnessing both national and local databases for prognostic studies on this type of cancer (17, 18). Historically, prognosis models for colon cancer have encapsulated various types without distinctly differentiating between left and right-sided colon cancers. Contemporary literature, however, underscores a significant disparity in the overall survival rates between right and left-sided colon cancers, with the former exhibiting notably lower survival rates (19–23). This suggests that crafting a separate prognostic model for right-sided colon cancer might enhance the accuracy of prognosis. Presently, the clinical and prognostic value of right-sided colon cancer within the broader context of colorectal cancer has not garnered ample attention. Consequently, our research seeks to establish a specialized nomogram model for the prognosis of right-sided colon cancer, aiming to aid physicians in risk stratification.

While the AJCC staging system is regarded as the benchmark for predicting the prognosis of colorectal cancer patients, our findings indicate potential inadequacies in its post-operative prognostic predictions. Currently, nomograms, based on multifactorial regressions which amalgamate various indicators and utilize calibrated lines to illustrate the interrelation of variables on a singular plane (24), dominate the clinical prognostic landscape. Due to their intuitive and user-friendly nature, nomograms play a pivotal role in shared decision-making between physicians and patients and are becoming increasingly prevalent in clinical settings. In fact, nomograms tailored for various tumors have showcased parity, and at times superiority, in prognostic evaluation compared to the traditional TNM staging (25, 26). However, it’s noteworthy that as the number of predictive factors in a nomogram increases, its complexity can escalate. In such scenarios, LASSO regression analysis emerges as an efficacious instrument to eliminate inconsequential predictive factors. LASSO, a regression technique predicated on penalizing the absolute values of regression coefficients, can, with appropriate adjustments, compress certain coefficients to zero, thereby expunging non-essential or minimally impactful covariates (27, 28). Thus, LASSO not only maintains the predictive precision of nomograms but as data accrues, the accuracy of these models is poised to amplify.

In our study, we harnessed the predictive capabilities of machine learning to develop a nomogram based on the SEER database to forecast postoperative overall survival in right-sided colon cancer patients. This nomogram exhibited superior predictive accuracy compared to the conventional TNM staging system. Predictive factors incorporated in the model include age, chemotherapy status, CEA, AJCC 7th Edition T, N, and M staging, summary stage, and liver metastasis. Our univariate and multivariate analyses revealed that gender, tumor location, and histological type were not independent prognostic factors for cancer survival (*P* > 0.05). Furthermore, ethnicity was determined to be non-influential on postoperative OS. This consistency in external validation results, coupled with the addition of clinically relevant prognostic factors, ensures the model’s applicability to the Chinese population.

Notably, the current AJCC staging guide omits age as a consideration. However, age stands as an independent predictor for both short-term and long-term postoperative mortality in cancer patients (29). Some studies have shown a rise in proximal colon tumors in patients aged <50 years and an association with expanding colon cancer screening practices, such as fecal occult blood tests and colonoscopies. Recent analyses indicate a decrease in colon cancer incidence among individuals aged 55-84 and a surge among those aged 20-55 (30–34). Lifestyle changes linked to Westernization, marked by shifts in dietary patterns over the past half-century, may explain these trends (35). In our study, patients aged <30 exhibited poorer outcomes than certain older cohorts, emphasizing that prevention and educational efforts should target younger demographics. The superior prognosis observed in the 40-60 age group can be attributed to their optimal physiological state. These findings advocate for the rationale behind initiating screenings at age 45 and routine screenings in individuals aged ≥50. As data on young right-sided colon cancer patients is sparse, further research is needed on personalized therapeutic strategies for this demographic. Patients aged >90 post-surgery have a markedly diminished 5-year OS compared to those aged <70, hinting at greater postoperative risks for the elderly, who also present with higher postoperative morbidity and mortality rates. These factors underscore the necessity for cautious therapeutic decisions, like surgical interventions, in elderly patients, and the imperative need for targeted management and continued research.

As an increasing number of researchers turn to the SEER and SEER-Medicare databases for outcome studies, we have identified several methods that amplify the potential of this data, deepening our understanding of right-sided colon cancer and enhancing patient care. The objective of future research is to refine staging and therapeutic techniques, thereby offering more personalized treatment options for right-sided colon cancer. To foster national improvements in care quality, it is essential to gain a profound insight into the care disparities among different regions and patient subgroups. Emphasizing primary prevention and early detection is particularly pivotal in addressing the challenges posed by an aging population and population growth.

## Conclusion

Based on the extensive SEER database, we developed and validated a line graph, serving as a convenient and reliable tool for individualized postoperative survival prediction in patients with right-sided colon cancer. This model, utilizing readily accessible data from clinical practice, delivers compelling individualized survival forecasts. Subsequent validation highlighted the model’s stellar performance in risk assessment. Consequently, our predictive tool empowers clinicians to accurately pinpoint high-risk patients, ensuring intensified follow-up and treatment strategies. Looking ahead, more prospective research is warranted to delve into survival prognostics for right-sided colon cancer patients.

## Data availability statement

The datasets presented in this study can be found in online repositories. The names of the repository/repositories and accession number(s) can be found in the article/**Supplementary Material**.

## Author contributions

TQ: Data curation, Formal analysis, Methodology, Software, Writing – original draft, Writing – review & editing. CY: Methodology, Supervision, Writing – review & editing. YD: Formal analysis, Project administration, Writing – review & editing. MZ: Data curation, Methodology, Writing – review & editing. XW: Formal analysis, Project administration, Writing – review & editing. XS: Writing – original draft, Writing – review & editing.
